# Determinants of post-stroke cognitive impairment and dementia: association with objective measures and patient-reported outcomes

**DOI:** 10.3389/fstro.2023.1190477

**Published:** 2023-08-23

**Authors:** Lara C. Oliveira, Anna K. Bonkhoff, Ana Ponciano, Carissa Tuozzo, Anand Viswanathan, Natalia S. Rost, Mark R. Etherton

**Affiliations:** ^1^J. Philip Kistler Stroke Research Center, Department of Neurology, Massachusetts General Hospital, Harvard Medical School, Boston, MA, United States; ^2^Stroke/Acute Neurology Neurovascular Therapeutics Development Unit, Biogen Inc., Cambridge, MA, United States

**Keywords:** ischemic stroke, post-stroke cognitive impairment, patient-reported outcome, stroke outcome, PROMs

## Abstract

**Background:**

Post-stroke cognitive impairment and dementia (PSCID) is a sequel of ischemic stroke (IS), highly prevalent and linked to poor long-term outcomes. Thus, early recognition of the clinical determinants of PSCID is urgent for identifying high-risk individuals who are susceptible to PSCID. And investigating objective measures of PSCID in relation to patient-reported outcome measures (PROMs) is essential for understanding the impact of IS. Here we identify the clinical determinants associated with PSCID and the relationship of PSCID to patient-reported outcomes in a population with IS.

**Methods:**

This was a cohort study. We enrolled 138 patients who were admitted to our hospital between February 2017 and February 2020, with IS and no pre-stroke diagnosis of dementia. Clinical variables were acquired on admission. At 3 months, patients underwent a follow-up evaluation including the Telephone Interview for Cognitive Status (TICS), modified Rankin scale (mRS), Barthel Index (BI), and PROMs, using the Patient-Reported Outcomes Measurement information System Global Health (PROMIS GH). MCI/Dementia was defined as a TICS score of <36. Regression analyses were used to identify clinical, functional, and patient-reported outcome determinants of the 3-month TICS score. Analyses were adjusted for age, stroke severity, and prior IS.

**Results:**

At follow-up, 113 participants (82%) were found to have MCI/Dementia. Patients with PSCID were more likely to be older, and at 3-months post-stroke they had lower rates of PROMIS GH T Mental (mean 47.69 vs. 52.13) and T Physical (mean 46.75 vs. 50.64). In multivariable linear regression analyses, increasing age (*β* = −0.07, *p* = 0.03) and Peripheral Artery Disease (PAD; *β* = −3.60, *p* = 0.03) were independently associated with a lower TICS score. Functional and patient-reported outcomes were also associated with worse TICS, including mRS ≥ 2, BI, T Mental, Global Mental, T Physical, and Global Physical in adjusted analyses. Individual components of PROMs were also associated with TICS, including quality of life, mental health, social satisfaction, and physical activities.

**Conclusions:**

In patients with IS, increased age and a pre-admission diagnosis of PAD are independently associated with worse objective measures of PSCID. Worse functional and patient-reported outcomes are also strongly linked to PSCID.

## Introduction

Ischemic stroke (IS) is a leading cause of long-term disability and cognitive dysfunction (Miller et al., 2010; Lozano et al., 2012; Feigin et al., 2014), and post-stroke cognitive impairment and dementia (PSCID) is a key determinant of poor long-term outcomes after stroke, causing a major burden to patients and health care systems (Rost et al., 2022). Several clinical factors associated with PSCID have been determined (Godefroy et al., 2018; Pendlebury and Rothwell, 2019), but despite the high prevalence of such impairment after stroke, identifying IS patients who are at risk for PSCID remains a challenge (Godefroy et al., 2018). Early recognition of the clinical determinants of PSCID will facilitate identification of high-risk IS patients and potentially enable individualized interventions, to reduce long-term disability.

Besides recognizing the clinical determinants of PSCID, understanding the interrelationship between objective measures of cognition and patient-reported outcomes could yield additional information on the individual impact of stroke and its heterogeneity. Traditional and objective measures for quantifying the functional status of patients who have suffered a stroke include the Modified Rankin Scale (mRS) and Barthel Index (BI), which constitute clinician-reported outcomes (Mahoney and Barthel, 1965; Banks and Marotta, 2007). However, these scales frequently do not illustrate the full range of outcomes experienced by stroke victims, mostly because they neglect other health domains (Katzan et al., 2018). As a consequence, interest has grown in collecting subjective health assessments directly from the patient, which are not filtered by anyone's interpretation and are outside the traditional clinician-reported measures (Katzan et al., 2017)—such patient-reported outcome measures (PROMs) thus represent a distinct opportunity to gauge stroke outcomes from the perspective of the patient (National Quality Forum, 2013; Reeves et al., 2018). A combined investigation of objective measures of cognitive function with PROMs provides additional insights into the impact of IS on individuals, and will better identify key clinical determinants associated with these outcomes. Moreover, the association between PROMs and PSCID is currently understudied, making our study's findings valuable in contributing to this field.

The Patient-Reported Outcomes Measurement Information System (PROMIS) was developed to measure health-related quality of life in various domains (Hahn et al., 2010). The International Consortium for Health Outcomes Measurement has recommended a set of measures specific to stroke that includes the PROMIS Global Health (PROMIS GH), a short form (Hays et al., 2009), that comprises 10 global questions that enable rapid calculation of mental and physical health scores (Hays et al., 2009). This form has the advantage that, when coupled with objective outcome measures, it evaluates the patients' opinion of their mental and physical health in relation to objective measures of post-stroke outcomes. Our objectives for this study were to: (1) identify the clinical determinants of PSCID; and (2) determine the association of PSCID with PROMs for physical and mental health in a population of IS patients with 3 month follow up.

## Materials and methods

### Study design and participants

**Figure 1:**
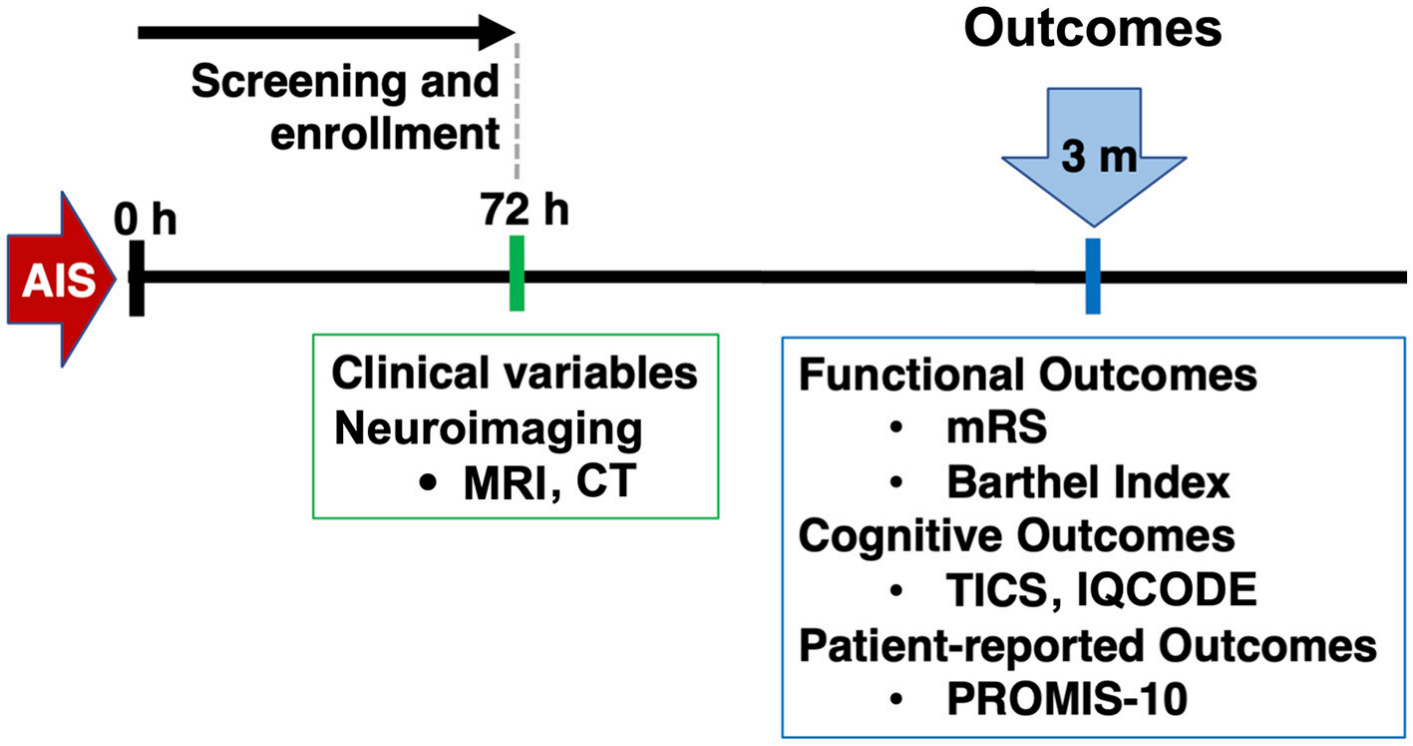
Study design. AIS, acute ischemic stroke; MRI, magnetic resonance imaging; CT, computed tomography; mRS, modified Rankin Scale; TICS, telephone interview for cognitive status; IQCODE, Informant Questionnaire on Cognitive Decline in the Elderly; PROMIS, patient-reported outcomes measurement information system.

This was a retrospective analysis of a single-center prospective cohort of IS. Inclusion criteria were: patients aged 18 or older, who presented to the Massachusetts General Hospital Emergency Department between February 2017 and February 2020, with a diagnosis of IS confirmed through magnetic resonance imaging (MRI) or computed tomography (CT). A notable exclusion criteria was pre-morbid diagnosis of dementia and lack of cognitive testing with the Telephone Interview for Cognitive Status (TICS) at the follow-up. Patients were initially assessed during a baseline visit to gather general clinical information. Subsequently, a single follow-up visit was conducted to collect outcome data (Figure 1).

### Clinical assessments

Clinical variables, including medical comorbidities and demographics, were acquired from each participant and from a review of medical records at the time of study enrollment. Admission stroke severity and pre-stroke disability were assessed by a trained neurologist, using the National Institutes of Health Stroke Scale score (NIHSS) and the modified Rankin scale (mRS), respectively (The National Institute of Neurological Disorders and Stroke rt-PA Stroke Study Group, 1995; Banks and Marotta, 2007). Functional outcomes were assessed between 3 and 6-months after stroke, by telephone interview with the patient or caregiver: these measures included mRS (Banks and Marotta, 2007), BI (Mahoney and Barthel, 1965), TICS (Zietemann et al., 2017), PROMIS GH questionnaires (Hays et al., 2009), and Informant Questionnaire on Cognitive Decline in the Elderly (IQ Code; Quinn et al., 2014). Raters for the test battery underwent a specialized training was performed by the senior author and principal investigator in this study (MRE).

The PROMIS GH short form is a 10-item form that allows for physical health and mental health subscores to be calculated from four items each (Hays et al., 2009). T scores for physical and mental health are generated by summing the raw scores of the individual responses and converting these to T scores for each domain. T scores distributions are standardized such that a score of 50 represents the average (mean) for the US general population, and the standard deviation (SD) around the mean is 10 points. Global Mental is the summing of Global 02 (quality of life), Global 04 (mental health), Global 05 (social satisfaction), and Global 10r (emotional problems). Global Physical corresponds to the summing of the responses for Global 03 (physical health), Global 06 (physical activities), Global 07rc (pain), and Global 08r (fatigue). In addition to those eight scores, the short form also contains assessments for the social health domain, addressing for patient's general health (Global 01) and social activities (Global 09r).

For follow-up assessments, we also gathered the IQCODE, which was validated in 1989. The IQCODE is a questionnaire in which an informant, typically a relative of the patient, is asked about the patient's cognitive changes during the past 10 years before onset of the stroke and rates them from 1 (much better) to 5 (much worse; Quinn et al., 2014). The score therefore informs about the cognitive performance before the stroke.

### Statistical analysis

We used R Version 4.1.1 (R Foundation for Statistical Computing, Vienna, Austria) for statistical analysis. TICS was dichotomized as < 36 for MCI/Dementia and ≥ 36 for normal cognitive status, based on the cut-off established in earlier work (Zietemann et al., 2017). IQCODE was also dichotomized for analysis as ≥ 3.3 (worsening of cognitive functions) and < 3.3 (no changes or improvement; Quinn et al., 2014). As appropriate, ANOVA and Chi square test were used to evaluate for baseline differences between the normal cognitive status and MCI/Dementia groups. No pre-stroke disability was defined as a pre-admission mRS of 0. Excellent functional outcome was defined as 3-month mRS < 2.

Linear regression analyses were performed to identify admission variables associated with the 3-month TICS score. Multivariable linear regression was then applied to identify stroke admission variables that were independently associated with the 3-month TICS, and linear regression analysis was conducted to evaluate the association between functional outcomes, PROMIS GH scores, and TICS score at 3 months. Avoiding potential confounding effects, analyses between TICS and 3-month outcomes were adjusted for age, admission stroke severity and prior stroke. Ordinal logistic regression was used to identify predictors of MCI/Dementia. All variables with an *a priori p* < 0.05 in the univariable regression analysis were included in the multivariable models. *P-*value of < 0.05 was considered statistically significant.

## Results

**Figure 2:**
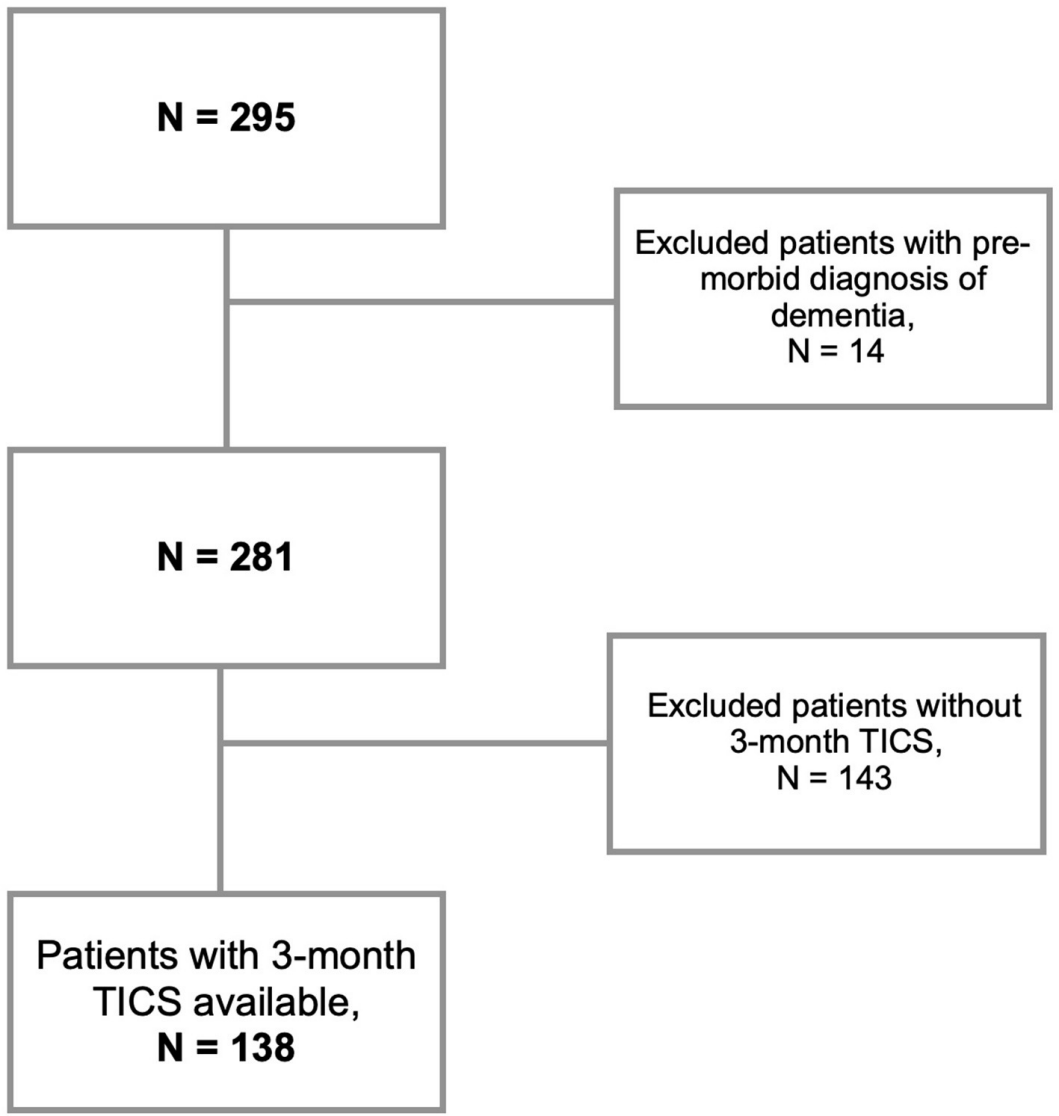
Patient selection. TICS, Telephone Interview for Cognitive Status.

All in all, the total number of screened acute IS patients was 295 patients. We here focused on the subset of patients (*N* = 138) with available follow-up TICS scores, and no past medical history of dementia (Figure 2). At 3-months, 82% of the patients (*N* = 113) had TICS < 36 (MCI/Dementia): *N* = 20 with TICS ≤ 28 (Dementia; Barber and Stott, 2004); and *N* = 93 with TICS between 29 and 35 (MCI; Zietemann et al., 2017).

Patients with MCI/Dementia were more likely to be older (65.89 vs. 59.41, *p* = 0.02) than were the individuals with TICS ≥ 36 (i.e., no cognitive impairment). We also saw a significant difference in admission stroke severity (NIHSS) between the two groups, but this difference was not relevant when considering median and IQR. No significant differences were seen in sex distribution, cardiovascular risk factors, or discharge mRS between the two groups. There was no significant difference with respect to discharge rehabilitation plans, whether to home, acute rehabilitation, nursing home, expired or other facility, between the study groups (Supplementary Table 1). At follow-up, patients with MCI/Dementia had lower rates of PROMIS GH T Mental (47.69 vs. 52.13, *p* = 0.02) and PROMIS GH T Physical (46.75 vs. 50.64, *p* < 0.05; Table 1).

**Table 1:** Baseline demographics of 138 ischemic stroke patients.

	**No cognitive impairment**	**Cognitive impairment**	
	**(TICS** ≥**36**, ***N*** = **25)**	**(TICS**<**36**, ***N*** = **113)**	
**Variables**			***P*** **-value**
Age [mean (SD)]	59.41 (13.62)	65.89 (11.91)	**0.02**
Female sex, *n* (%)	11 (44.0)	48 (42.5)	1.00
White race, *n* (%)	24 (96.0)	105 (94.6)	1.00
No pre-stroke disability, *n* (%)	19 (79.2)	89 (80.2)	1.00
**Medical history**, ***n*** **(%)**			
Hypertension	18 (75.0)	86 (77.5)	1.00
Hyperlipidemia	13 (56.5)	79 (71.2)	0.26
Diabetes mellitus II	5 (20.8)	28 (25.2)	0.85
Obese	19 (79.2)	90 (80.4)	1.00
CAD	4 (16.7)	23 (20.7)	0.87
PAD	1 (4.2)	7 (6.3)	1.00
Atrial fibrillation	3 (12.5)	11 (9.9)	0.99
Heart failure	4 (16.7)	7 (6.3)	0.20
Prior IS/TIA	3 (13.0)	19 (17.6)	0.82
**Admission data**			
NIHSS [median (IQR)]	2.000 (1.000, 7.000)	2.000 (1.000, 5.000)	**< 0.05**
IVtPA, *n* (%)	7 (58.3)	11 (37.9)	0.39
Discharge mRS 0–1, *n* (%)	6 (25)	37 (33.3)	0.58
**3-month outcomes**			
mRS 0–1, *n* (%)	11 (44.0)	44 (39.3)	0.83
Barthel index [mean (SD)]	97.60 (4.81)	92.88 (14.23)	0.10
IQCODE ≥ 3.3, *n* (%)	1 (4.0)	18 (16.2)	0.20
PROMs T Mental [mean (SD)]	52.13 (8.79)	47.69 (8.06)	**0.02**
PROMs T Physical [mean (SD)]	50.64 (9.31)	46.75 (8.38)	**< 0.05**

CAD, coronary artery disease; IQCODE, Informant Questionnaire on Cognitive Decline in the Elderly; IS, ischemic stroke; IV, intravenous; MCI, mild cognitive impairment; mRS, modified Rankin Scale; NIHSS, National Institutes of Health Stroke Scale; PROMs, patient-reported outcome measures; PAD, peripheral artery disease; TIA, transient ischemic attack.

P-values < 0.05 are in bold.

In univariable linear regression analysis, we observed that increasing age (*β* = −0.09, *p* = 0.01), hyperlipidemia (*β* = −2.11, *p* = 0.02), and peripheral artery disease (PAD; *β* = −4.23, *p* = 0.01) were associated with a lower TICS score. In multivariable linear regression analysis, increasing age (*β* = −0.07, *p* = 0.03) and PAD (*β* = −3.60, *p* = 0.03) were independently associated with lower 3-month TICS score (Table 2).

**Table 2:** Linear regression for determinants of 3-month TICS in 138 IS patients.

	**Univariable analysis**			**Multivariable analysis**		
**Predictors**	**Estimates**	**CI**	***P*** **-value**	**Estimates**	**CI**	***P*** **-value**
Age	−0.09	−0.15, −0.03	**0.01**	−0.07	−0.14, −0.01	**0.03**
Female sex	0.01	−1.61, 1.64	0.99			
No pre-stroke disability	1.16	−0.87, 3.19	0.26			
Hypertension	−1.41	−3.33, 0.52	0.15			
Hyperlipidemia	−2.11	−3.82, −0.39	**0.02**	−1.23	−2.96, 0.50	0.16
Diabetes mellitus II	−1.57	−3.45, 0.31	0.10			
Obese	0.55	−1.48, 2.57	0.59			
PAD	−4.23	−7.60, −0.85	**0.01**	−3.60	−6.88, −0.31	**0.03**
Atrial fibrillation	−0.63	−3.30, 2.04	0.64			
Heart failure	0.36	−2.62, 3.34	0.81			
Prior IS/TIA	−0.33	−2.55, 1.89	0.77			
NIHSS	0.00	−0.14, 0.15	0.98			
IV tPA	2.57	−0.10, 5.23	0.06			
Discharge mRS ≥ 2	−1.38	−3.11, 0.36	0.12			

PAD, peripheral artery disease; IS, ischemic stroke; TIA, transient ischemic attack; NIHSS, National Institutes of Health Stroke Scale; IV, intravenous; mRS, modified Rankin Scale.

P-values < 0.05 are in bold.

Next, we evaluated the relationship between 3-month TICS and functional and patient-reported outcomes. A lower 3-month TICS score was associated with worse functional outcomes (mRS ≥ 2: *β* = −2.75, *p* < 0.01; BI: *β* = 0.17, *p* < 0.001), and worse scores for patient-reported T Mental (*β* = 0.15, *p* < 0.001) and T Physical (*β* = 0.11, *p* < 0.01. We also found a significant association between IQCODE ≥ 3.3 and worse TICS (*β* = −2.94, *p* < 0.01). In adjusted analysis for age, NIHSS, and prior stroke, all associations remained consistent (Table 3).

**Table 3:** Association between TICS and 3-month outcomes.

**Outcomes**	**Estimates**	**CI**	***P*-value**
mRS ≥ 2	−2.75	−4.34, −1.17	**< 0.01**
*Adjusted for age, NIHSS and prior IS*	−2.62	−4.25, −0.99	**< 0.01**
Barthel index	0.17	0.12, 0.23	**< 0.001**
*Adjusted for age, NIHSS and prior IS*	0.17	0.12, 0.23	**< 0.001**
IQCODE ≥ 3.3	−2.94	−4.87, −1.01	**< 0.01**
*Adjusted for age, NIHSS and prior IS*	−3.10	−4.95, −1.24	**< 0.01**
T Mental	0.15	0.07, 0.23	**< 0.001**
*Adjusted for age, NIHSS and prior IS*	0.15	0.07, 0.23	**< 0.001**
Global Mental	0.40	0.18, 0.61	**< 0.001**
*Adjusted for age, NIHSS and prior IS*	0.39	0.18, 0.61	**< 0.001**
T Physical	0.11	0.03, 0.19	**< 0.01**
*Adjusted for age, NIHSS and prior IS*	0.10	0.02, 0.18	**0.02**
Global Physical	0.32	0.08, 0.56	**< 0.01**
*Adjusted for age, NIHSS and prior IS*	0.30	0.04, 0.55	**0.02**

mRS, modified Rankin Scale; NIHSS, National Institutes of Health Stroke Scale; IS, ischemic stroke; IQCODE, Informant Questionnaire on Cognitive Decline in the Elderly.

P-values < 0.05 are in bold.

Individual items from the PROMIS GH form were also individually associated with the 3-month TICS. For mental health, all four sub-items, except for Global 10r (emotional problems), were determinants of the 3-month TICS score. Higher scores for quality of life (Global 2—*β* = 1.30, *p* < 0.001), mental health (Global 04—*β* = 0.76, *p* = 0.03) and social satisfaction (Global 05—*β* = 1.26, *p* < 0.001), as well as Global Mental (*β* = 0.40, *p* < 0.001), which is the composite of these four parameters, were all associated with a better 3-month TICS score. As for physical health, only Global06 (physical activities; *β* = 1.10, *p* < 0.001) and Global Physical (*β* = 0.32, *p* < 0.01) were associated with 3-month TICS score. The PROMIS GH components Global 01 and Global 09r, which represent general health and social activities respectively, were also associated with 3-month TICS score (Table 4).

**Table 4:** Association between TICS and PROMIS Global Health items.

	**Univariable analysis**		
**PROMIS GH**	**Estimates**	**CI**	***P*** **-value**
Global 01: general health	1.08	0.35, 1.81	**< 0.01**
Global 02: quality of life	1.30	0.66, 1.93	**< 0.001**
Global 03: physical health	0.50	−0.27, 1.27	0.20
Global 04: mental health	0.76	0.07, 1.44	**0.03**
Global 05: social satisfaction	1.26	0.62, 1.90	**< 0.001**
Global 06: physical activities	1.10	0.50, 1.71	**< 0.001**
Global 07rc: pain	0.45	−0.26, 1.15	0.21
Global 08r: fatigue	0.58	−0.23, 1.39	0.16
Global 09r: social activities	1.01	0.35, 1.67	**< 0.01**
Global 10r: emotional problems	0.42	−0.27, 1.10	0.24

TICS, Telephone Interview for Cognitive Status; PROMIS GH, Patient-Reported Outcome Measurement Information System Global Health.

P-values < 0.05 are in bold.

To further investigate the clinical determinants associated with PROMs, we then used linear regression analysis to determine predictors of 3-month patient-reported physical and mental outcomes. PAD (*β* = −6.65, *p* = 0.02) and discharge mRS ≥ 2 (*β* = −3.86, *p* = 0.01) were associated with lower 3-month PROMIS GH T mental in univariable model. In multivariable analysis, discharge mRS ≥ 2 (*β* = −3.32, *p* = 0.03) was an independent predictor of lower 3-month T Mental scores (Table 5).

**Table 5:** Linear regression for determinants of 3-month T Mental score.

	**Univariable analysis**			**Multivariable analysis**		
**Predictors**	**Estimates**	**CI**	***P*** **-value**	**Estimates**	**CI**	***P*** **-value**
Age	0.05	−0.07, 0.17	0.39			
Female sex	−2.60	−5.49, 0.28	0.08			
No pre-stroke disability	3.23	−0.35, 6.81	0.08			
Hypertension	−2.18	−5.68, 1.33	0.22			
Hyperlipidemia	−0.99	−4.11, 2.13	0.53			
Diabetes mellitus II	−2.73	−6.10, 0.64	0.11			
Obese	1.44	−2.20, 5.09	0.43			
PAD	−6.65	−12.34, −0.96	**0.02**	−5.52	−11.23, 0.20	0.06
Atrial fibrillation	0.93	−3.82, 5.67	0.70			
Heart failure	−0.40	−5.48, 4.69	0.88			
Prior IS/TIA	−3.05	−6.82, 0.72	0.11			
NIHSS	−0.23	−0.49, 0.03	0.09			
Discharge mRS ≥ 2	−3.86	−6.90, −0.83	**0.01**	−3.32	−6.38, −0.26	**0.03**
**3-month outcome**						
mRS ≥ 2	−7.76	−10.38, −5.13	**< 0.001**			
Barthel index	0.28	0.16, 0.39	**< 0.001**			
IQCODE ≥ 3.3	−9.23	−13.15, −5.30	**< 0.001**			

PAD, peripheral artery disease; IS, ischemic stroke; TIA, transient ischemic attack; NIHSS, National Institutes of Health Stroke Scale; IV, intravenous; mRS, modified Rankin Scale; IQCODE, Informant Questionnaire on Cognitive Decline in the Elderly.

P-values < 0.05 are in bold.

In univariable linear regression analysis for predictors of 3-month PROMIS GH T Physical, female sex (*β* = −4.40, *p* < 0.01), no pre-stroke disability (*β* = 7.63, *p* < 0.001), and discharge mRS ≥ 2 (*β* = −4.02, *p* = 0.01) were associated with lower T Physical scores. In multivariable analysis, female sex (*β* = −3.21, *p* = 0.03), no pre-stroke disability (*β* = 6.72, *p* < 0.001), and discharge mRS ≥ 2 (*β* = −3.47, *p* = 0.02) remained independently associated with worse T Physical scores (Table 6).

**Table 6:** Linear regression for determinants of 3-month T Physical score.

	**Univariable analysis**			**Multivariable analysis**		
**Predictors**	**Estimates**	**CI**	***P*** **-value**	**Estimates**	**CI**	***P*** **-value**
Age	0.01	−0.11, 0.14	0.82			
Female sex	−4.40	−7.40, −1.40	**< 0.01**	−3.21	−6.07, −0.35	**0.03**
No pre-stroke disability	7.63	4.06, 11.20	**< 0.001**	6.72	3.21, 10.23	**< 0.001**
Hypertension	−1.25	−4.90, 2.40	0.50			
Hyperlipidemia	−0.37	−3.65, 2.91	0.82			
Diabetes mellitus II	−2.44	−5.99, 1.11	0.18			
Obese	−1.16	−4.99, 2.67	0.55			
PAD	−3.96	−10.02, 2.10	0.20			
Atrial fibrillation	−3.04	−8.00, 1.92	0.23			
Heart failure	−2.58	−7.91, 2.74	0.34			
Prior AIS/TIA	−3.37	−7.30, 0.57	0.09			
NIHSS	−0.22	−0.50, 0.05	0.11			
Discharge mRS ≥ 2	−4.02	−7.18, −0.85	**0.01**	−3.47	−6.42, −0.51	**0.02**
**3-month outcome**						
mRS ≥ 2	−9.08	−11.75, −6.41	**< 0.001**			
Barthel index	0.35	0.23, 0.47	**< 0.001**			
IQCODE ≥ 3.3	−9.74	−13.70, −5.78	**< 0.001**			

PAD, peripheral artery disease; IS, ischemic stroke; TIA, transient ischemic attack; NIHSS, National Institutes of Health Stroke Scale; IV, intravenous; mRS, modified Rankin Scale; IQCODE, Informant Questionnaire on Cognitive Decline in the Elderly.

P-values < 0.05 are in bold.

Lastly, we evaluated the determinants of MCI/Dementia at 3 months post-stroke, based on the dichotomized TICS score. In the mental domain, T Mental (OR 0.94; 95% CI, 0.88–0.99), Global Mental (OR 0.85; 95% CI, 0.72–0.98), Global 02 (quality of life; OR 0.42; 95% CI, 0.23–0.69), and Global 05 (social satisfaction; OR 0.60; 95% CI, 0.36–0.95) were predictors of MCI/Dementia. As for the physical assessments, the independent determinants for MCI/Dementia were T Physical (OR 0.95; 95% CI, 0.90–1.00) and Global 08r (fatigue; OR 0.57; 95% CI, 0.−32 – 0.98; Supplementary Table 2).

The full list of baseline characteristics of the here included 138 patients with 3-month TICS scores and the 143 patients without available TICS scores are described in the supplement. Overall, patients with 3-month TICS available were more likely to be younger (64.71 vs. 69.23, *p* < 0.01), had higher rates of no pre-stroke disability (80 vs. 61%, *p* 0.001), lower median NIHSS at admission (2 vs. 4, *p* 0.001), and lower rates of discharge mRS ≥ 2 (68.1 vs. 88.9%, *p* < 0.001; Supplementary Table 3). Given the higher NIHSS and mRS in the excluded patients, we believe the frequency of MCI/Dementia would be even higher in the full sample and our results would likely remain relevant.

## Discussion

Our study aimed to identify the clinical determinants of PSCID and determine the association of PSCID with PROMs for physical and mental health in a population of IS patients with 3 month follow up. In our population, a substantial proportion (82%) of patients presented with MCI/Dementia 3 months after stroke. This corroborates reports of a high overall occurrence (76–92%) of cognitive impairment up to 3 months after stroke in one or more cognitive domains in prior publications (Lesniak et al., 2008; Middleton et al., 2014; Jokinen et al., 2015).

Our findings highlight the novelty of the relationship between PSCID and PROMs. Moreover, following stroke, PROMs are strongly linked to functional and cognitive outcomes. We also report on the clinical determinants of PSCID demonstrating that increased age and peripheral artery disease are independently associated with worse cognitive performance at our follow-up. To mitigate potential confounding effects of pre-existing cognitive impairment, we excluded patients with a documented pre-stroke diagnosis of dementia based on their medical records. This exclusion aimed to minimize the influence of pre-existing cognitive deficits on our findings. However, we acknowledge that accurately determining the pre-stroke cognitive status of participants remains a limitation in our study.

Furthermore, we highlight the potential of PAD in identifying patients at higher risk of cognitive impairment and functional disability. Early identification allows healthcare professionals to initiate timely interventions and support systems to address the specific needs of these individuals. By implementing proactive measures, such as cognitive rehabilitation programs or referral to specialized services, healthcare providers can help optimize cognitive recovery and promote better long-term outcomes. However, it is important to acknowledge that the association between PAD and PSCID was based on a small subset of the overall sample, which represents a limitation in terms of generalizability and statistical power. As such, there is need for caution in interpreting these findings and further research with larger sample sizes is warranted to validate and expand upon these preliminary results.

As a marker of generalized atherosclerosis, PAD results from pathophysiological mechanisms that are also implicated in the development of cognitive decline (Yang et al., 2020). PAD affects the cardiovasculature in general and therefore, increases the risk for vascular-related pathologies, such as vascular dementia (Yang et al., 2020). Consistent with our findings, prior studies described that PAD, which is often associated with white matter lesions and cerebral atrophy, was also associated with cognitive decline (van der Veen et al., 2014). It is a known risk factor of post-stroke dementia and cognitive impairment (Houghton et al., 2021). Nevertheless, the prevalence of PSCID in patients with PAD and stroke remains poorly described (Houghton et al., 2021), and recognition of the clinical determinants of PSCID is urgent for identifying high-risk individuals susceptible to PSCID.

IS patients with a low TICS score at 3 months are more likely to have functional disability and poor patient-reported mental and physical health outcomes. The TICS is a Global Mental status test, with excellent sensitivity and specificity in identifying participants with cognitive impairment such as PSCID (Knopman et al., 2010). Cognitive domains measured by the TICS include orientation, concentration, short-term memory, language, praxis and mathematical skills (Knopman et al., 2010). A major advantage of using the TICS test for post-stroke patients is that, unlike the Mini-Mental State Exam (MMSE), TICS can be administered to individuals with severe visual or motor impairments (Knopman et al., 2010). In addition, as a phone-based assessment of cognitive status, it offers advantages in simplifying data acquisition and reducing loss to follow up because of possible challenges with performing an in-person assessment (Desmond et al., 1994). Previous research also supports the TICS as a reliable and valid method that provides accurate information regarding cognitive function in post-stroke patients (Desmond et al., 1994; Barber and Stott, 2004).

Further, age, stroke severity, and previous stroke could be potential confounding factors to our results. However, associations between worse TICS and worse mRS, BI, IQCODE, and PROMs remained significant after adjusting for age, NIHSS, and prior IS. Also, stroke severity has been described as a significant risk factor in the occurrence of PSCID (Pendlebury and Rothwell, 2019). However, the median NIHSS of our population was only 2. Our results therefore highlight that even minor stroke patients are at risk for PSCID and should be taken into consideration for preventative measures.

We also report on the relationship between PROMs of physical and mental health and functional and cognitive outcomes. Lower TICS scores were associated with worse PROMIS GH subitems and functional objective outcomes, represented here by the mRS and the Barthel Index. The PROMIS GH questionnaire has previously been validated in patients with IS (Katzan and Lapin, 2018). In our results, worse T and global scores for mental and physical health, as well as isolated components that stand for quality of life, general and mental health, social satisfaction, and social and physical activities, were independently associated with worse cognitive status. Several studies explored the association between cognitive disorders after stroke with different measures for low health-related quality of life (Hochstenbach et al., 2001; Sturm et al., 2004; Nys et al., 2006; Cumming et al., 2014). Nevertheless, we report on the novelty of using a distinct questionnaire (i.e., PROMIS GH) to assess diverse domains of quality of life and their relationship with cognitive impairment after stroke. By integrating PROMs into the evaluation of PSCID, healthcare professionals can gain a more comprehensive understanding of patients' cognitive function and its impact on their daily lives. This information can then be used to develop individualized rehabilitation strategies and interventions aimed at improving cognitive function and overall quality of life for stroke victims.

While our study focused on identifying associations rather than elucidating underlying mechanisms, we can speculate on some possible explanations for the link between TICS scores with functional disability and PROMs. Cognitive impairment following an ischemic stroke can directly impact an individual's ability to perform daily activities. Cognitive dysfunction, such as difficulties with memory, attention, and executive functions, may hinder an individual's capacity to carry out tasks essential for independent living and self-care. This, in turn, could contribute to functional disability and adversely affect patient-reported mental and physical health outcomes. Additionally, cognitive impairment may also have indirect effects on mental and physical health outcomes. The cognitive challenges experienced by stroke survivors could lead to increased psychological distress, including feelings of frustration, depression, and anxiety. These emotional factors can have a negative impact on overall mental wellbeing and potentially influence physical health as well. However, more research is needed to explore these physiological mechanisms in greater detail and confirm their relevance in the context of our study findings.

Moreover, patient-reported outcomes provide additional information on health status in stroke patients in the ambulatory setting, since clinician-reported measures may not fully represent the global health status (Katzan et al., 2017). Rehabilitation strategies for stroke survivors encompass understanding outcomes meaningful to them. Identifying and addressing improvements in these outcomes is essential for patients' optimal recovery (Salinas et al., 2016). Previous publications have shown a wide variability of outcomes for physical, social and cognitive function that is not always captured by the mRS (Katzan et al., 2017; Reeves et al., 2018; Price-Haywood et al., 2019). Also, PROMs have been shown to be reliable and valid across stroke subtype and disability level (mRS < 2 vs. ≥2) suggesting that this questionnaire can be applied to a broad spectrum of stroke patients (Katzan and Lapin, 2018). Importantly, 97.1% (*N* = 134) of the patients answered the questionnaires personally and only one had caregiver assistance, indicating remarkable reliability in our results of patients' perception of their symptoms.

Lastly, we explored the association of patient-reported mental and physical health with distinct determinants for these domains. For T Mental, discharge mRS of ≥ 2 was a determinant of worse scores, while for T Physical, associations were observed for female sex, absence of pre-stroke disability and mRS of ≥ 2 at discharge. These results provide important insights into the complex interplay between physician-collected and patient-reported outcomes. It is widely encouraged to investigate depression, anxiety and fatigue in post-stroke patients because they are highly prevalent and associated with poor functional and cognitive outcomes (West et al., 2010; Mitchell et al., 2017). Measures that capture these domains from the viewpoint of the patient are especially important during stroke recovery, in order to provide adequate post-stroke care (Godefroy et al., 2018; Price-Haywood et al., 2019). Screening for post-stroke mood disorders in combination with assessments of PROMs may enhance a more individualized assessment of stroke impact and outcome. Although stroke care teams are familiar with PROMs questionnaires, their use to improve the quality of care of patients is still uncommon, especially with regard to comparing its association with objective cognitive evaluation (Rumsfeld et al., 2013; Cella et al., 2015; Price-Haywood et al., 2019). In the context of PSCID, incorporating PROMs could thus be an important strategy that supplements traditional clinician-reported outcome measures. Use of both objective and patient-reported measures could therefore be effective for assessing quality of life and care of these patients.

Our study has a few limitations. First, while enhancing the feasibility, our follow-up evaluation was phone-based only and, unfortunately, this choice resulted in a large loss to follow-up (*N* = 143 that could not be reached via phone). In addition, a single phone interview might not be sufficient to fully capture patients' outcomes. Yet, we believe that relevant outcome measures can be obtained via phone calls as prior studies have shown a high correlation and feasibility for phone-based outcomes assessments (Cooray et al., 2015). Moreover, some patients were not available for the 3-month interview, and a timeframe from 3 to 6 months for reassessment was necessary, which could affect patient functional recovery if more time to rehabilitation at time of our approach. While rehabilitation programs play a crucial role in the recovery process and could be a influencing factor in these results, there was no significant difference in discharge rehabilitation plans among our study groups (TICS ≥ 36 vs. TICS < 36). Furthermore, according to the Copenhagen Stroke Study, the majority of stroke patients (95%) reach their best outcome score between 8.5 and 13 weeks after the stroke (Jørgensen et al., 1995). This information provides reassurance that a substantial portion of our study population would have already achieved near maximum recovery by the time of the outcomes assessment. Furthermore, the study also found that milder strokes tend to recover more quickly (Jørgensen et al., 1995), which is applicable to our analysis.

Second, we do not have sufficient data on depressive symptoms in our cohort that would allow for a formal diagnosis of depression. While we overall paid great care to obtain a comprehensive range of clinical information and outcome measures, we did not have the time capacity to additionally perform an in-depth assessment of patients with respect to depressive symptoms, neither at baseline, nor at follow-up. In particular, applying a specific questionnaire to assess depression would require a more specialized interview, that may not be feasible within the Framework of a phone-based contact. However, the PROMs we collected contain questions about self-perception regarding mental and social health, such as quality of life, mental health, social satisfaction, and social activities. Therefore, while these PROMs may not allow for a formal diagnosis of depression, our use of PROMs may offer additional insight into patients' perspectives of their mental and physical health. Further, all of these PROMs focused on mental health were significantly associated with worse TICS score.

Third, it may have been more ideal to investigate patients with MCI and dementia separately, rather than merged into one group. However, such an approach was not feasible given that this was a single center study with a limited number of participants. Considering that both diagnoses share similarities in pathomechanisms and, therefore risks predictors, we treated patients both with MCI and dementia as PSCID. Altogether, this approach is in line with a recent review by Rost et al. (2022). In addition, according to our study protocol Barthel index was a follow-up outcome only and data on admission BI is not available. However, data on pre-morbid mRS was obtained at baseline and analyses between TICS and 3-month outcomes, after adjusting for pre-stroke disability, remained significant.

Fourth, our study design was specifically focused on IS patients. We made this decision considering the prevalence and impact of cognitive impairment in IS populations, as well as the need for a more targeted analysis to assess the determinants associated with PSCID in this specific subgroup. Investigating associations between cognitive outcomes in hemorrhagic stroke patients is warranted. Moreover, data on stroke prevention medications or mood disorder treatments was not available. Yet, all patients admitted to our center are guaranteed to have an outpatient follow-up appointment that ensures that the best medical therapy is provided for the full range of symptoms when indicated. Also, the population was restricted to a single Comprehensive Stroke Center, which could affect the ability to generalize. However, given the large geographic catchment area of our stroke center as well as the telestroke network (New England), we believe that the study sample is representative of the regional population. And lastly, our study was an analysis of patients with relatively minor IS (median NIHSS 2): future studies will need to include more severe stroke populations, such as patients with large-vessel occlusions, to ensure the generalizability of our findings.

## Conclusion

Increasing age, pre-admission diagnosis of PAD, and patient-reported outcomes are independently associated with worse objective measures of PSCID. Incorporating PROMs into IS outcome measures may offer additional insight into the individual impact of IS on post-stroke outcomes and quality of life.

## Data availability statement

The raw data supporting the conclusions of this article will be made available by the authors, without undue reservation.

## Ethics statement

The studies involving human participants were reviewed and approved by Institutional Review Board (IRB—IRB2019P001189) and uses phenotypic and imaging data collected under IRB2013P000494. The patients/participants provided their written informed consent to participate in this study.

## Author contributions

LO was responsible for all the statistical analysis, interpretation of data, and wrote the manuscript draft. ME conceived the study design and revised the manuscript critically. CT was involved in patient recruitment and follow-up assessments. NR, AV, AB, and AP reviewed and edited the manuscript. All authors approved the final version of the manuscript.
