# Supplementary material for: Determinants of post-stroke cognitive impairment and dementia: association with objective measures and patient-reported outcomes
Source: Front Stroke. 2023 Aug 23;2:1190477. doi: 10.3389/fstro.2023.1190477 (PMC12802749; doi:10.3389/fstro.2023.1190477)
Supplement: Supplementary file 1 [file Data_Sheet_1.docx]

**SUPPLEMENTAL MATERIAL**

Table 1. Discharge Rehabilitation Plan of 138 Ischemic Stroke Patients

|  | **No Cognitive Impairment**  **(TICS ≥ 36, N = 25)** | **Cognitive Impairment**  **(TICS < 36, N = 113)** |  |
| --- | --- | --- | --- |
| **Discharge Rehabilitation Plan** |  |  | ***P value*** |
| Home | 14 (58.3) | 71 (64.0) | 0.78 |
| Acute Rehabilitation | 9 (37.5) | 36 (32.4) | 0.81 |
| Nursing Home | 0 (0.0) | 1 (0.9) | 1.00 |
| Expired | 0 (0.0) | 0 (0.0) | NaN |
| Other | 1 (4.2) | 3 (2.7) | 1.00 |

*TICS indicates telephone interview for cognitive status; NaN, not a number.*

Table 2. Logistic Regression of MCI/Dementia (TICS < 36) and PROMs scores at 3-months

|  | **Univariable Analysis** | | |
| --- | --- | --- | --- |
| **PROMs** | ***Odds Ratios*** | **CI** | ***P value*** |
| Global 01 | 0.74 | 0.45 – 1.19 | 0.22 |
| Global 09r | 0.62 | 0.38 – 0.98 | 0.05 |
| **Mental** |  |  |  |
| T Mental | 0.94 | 0.88 – 0.99 | **0.02** |
| Global Mental | 0.85 | 0.72 – 0.98 | **0.03** |
| Global 02 | 0.42 | 0.23 – 0.69 | **<0.01** |
| Global 04 | 0.84 | 0.52 – 1.30 | 0.44 |
| Global 05 | 0.60 | 0.36 – 0.95 | **0.04** |
| Global 10r | 0.91 | 0.58 – 1.41 | 0.69 |
| **Physical** |  |  |  |
| T Physical | 0.95 | 0.90 – 1.00 | **<0.05** |
| Global Physical | 0.86 | 0.72 – 1.01 | 0.07 |
| Global 03 | 0.80 | 0.49 – 1.30 | 0.37 |
| Global 06 | 0.62 | 0.37 – 0.98 | 0.06 |
| Global 07rc | 0.87 | 0.54 – 1.36 | 0.57 |
| Global 08r | 0.57 | 0.32 – 0.98 | **<0.05** |

*MCI indicates mild cognitive impairment; TICS, Telephone Interview for Cognitive Status; and PROMs indicates patient-reported outcome measures. P values < 0.05 are in bold.*

Table 3. Baseline Demographics of 138 Ischemic Stroke Patients

|  | **3-month TICS** | |  |
| --- | --- | --- | --- |
| **Variables** | **Not available**  **N = 143** | **Available**  **N = 138** | ***P value*** |
| Age (mean (SD)) | 69.23 (14.3) | 64.71 (12.44) | **<0.01** |
| Female Sex, n (%) | 57 (39.9) | 59 (42.8) | 0.71 |
| White Race, n (%) | 122 (87.1) | 129 (94.9) | **0.04** |
| No Pre-Stroke Disability, n (%) | 83 (61.0) | 108 (80.0) | **0.001** |
| **Medical History, n (%)** |  |  |  |
| Hypertension | 100 (74.1) | 104 (77.0) | 0.67 |
| Hyperlipidemia | 79 (58.1) | 92 (68.7) | 0.09 |
| Diabetes Mellitus II | 34 (25.0) | 33 (24.4) | 1.00 |
| Obese | 103 (75.7) | 109 (80.1) | 0.47 |
| CAD | 39 (29.1) | 27 (20.0) | 0.11 |
| PAD | 8 (5.9) | 8 (5.9) | 1.00 |
| Atrial Fibrillation | 30 (22.6) | 14 (10.4) | 0.01 |
| Heart Failure | 17 (12.6) | 11 ( 8.1) | 0.32 |
| Prior IS/TIA | 25 (18.4) | 22 (16.8) | 0.86 |
| **Admission Data** |  |  |  |
| NIHSS (median (IQR)) | 4.000 (2.000, 10.000) | 2.000 (1000, 5.250) | **0.001** |
| IVtPA, n (%) | 24 (50.0) | 18 (43.9) | 0.72 |
| Discharge mRS ≥2, n (%) | 120 (88.9) | 92 (68.1) | **<0.001** |

*CAD indicates coronary artery disease; TICS, telephone interview for cognitive status; IS, ischemic stroke; IV, intravenous; mRS, modified Rankin Scale; NIHSS, National Institutes of Health Stroke Scale;* ***PAD****, peripheral* ***artery*** *disease; and TIA, transient ischemic attack. P values < 0.05 are in bold.*
